# A control system analysis of the dynamic response of *N*-methyl-D-aspartate glutamate receptors to alcoholism and alcohol withdrawal

**DOI:** 10.1186/s12976-015-0004-3

**Published:** 2015-05-16

**Authors:** Carlos A Gutierrez, Mary M Staehle

**Affiliations:** Department of Chemical Engineering, Rowan University, 201 Mullica Hill Road, 08028 Glassboro, NJ USA; Department of Biomedical Engineering, Rowan University, 201 Mullica Hill Road, 08028 Glassboro, NJ USA

**Keywords:** Alcohol dependence, Alcohol withdrawal, Control system modeling, NMDA receptors, Glutamatergic signaling

## Abstract

**Background:**

*N*-methyl-D-Aspartate (NMDA) and its receptors (NMDAR) play a critical role in glutamatergic neurotransmission. Ethanol molecules inhibit these receptors, and if the brain is exposed to ethanol chronically, NMDA-induced glutamatergic changes can result in physical dependence to ethanol in order to sustain normal brain function. In these cases, removal of ethanol from the system results in excitotoxic withdrawal. One compensatory mechanism the brain uses to regulate extracellular glutamate concentration is modulating the number of NMDARs at the synapse. Previous work has shown that the number of functional NMDARs at the synapse can be changed by three mechanisms: additional receptors can be synthesized and inserted, receptors can be recruited to the synapse from extrasynaptic regions, or the functionality of existing receptors can be modified.

**Methods:**

In this study, we consider the dynamic relocation control of NMDARs in response to chronic alcoholism and withdrawal. Specifically, we (1) propose and construct a mathematical model of the relocation control as a negative feedback system with an explicit set point, (2) investigate the effect of various ethanol consumption and withdrawal profiles on the NMDAR population, and (3) propose and calculate quantitative measures for the extent of withdrawal based on modeled NMDAR populations.

**Results:**

A relocation-only model with an explicit set point was developed. The model was shown to apply across a wide range of controller parameters. The results suggest that withdrawal severity does not depend upon the dynamics involved in the development of dependence, and that regulating the blood alcohol level throughout the progression of withdrawal can minimize excitotoxic withdrawal symptoms.

**Conclusions:**

The negative feedback control system produced characteristic behaviors of NMDAR populations in response to simulations of alcohol dependence and abrupt withdrawal. The model can also predict the severity of excitotoxic withdrawal following various alcohol consumption and/or withdrawal patterns in order to generate testable hypotheses regarding ameliorating withdrawal.

## Background

Chronic alcoholism develops when individuals regularly consume pharmacologically significant quantities of ethanol over an extended period of time. The constant presence of ethanol leads to homeostatic adaptations in the brain and its neurotransmitter systems to compensate for the neurological effects of ethanol. Given enough time, this can lead to a state where the brain is physically dependent upon ethanol to function [1]; that is to say that the brain does not function appropriately without a high concentration of ethanol in the bloodstream. Once dependence has developed, if the individual ceases to consume alcohol, they may experience symptoms of withdrawal. These symptoms imply a dysregulation of the body’s internal equilibrium or homeostasis, and can manifest both physiologically and emotionally.

Studies have implicated neuroexcitatory transmitter systems as part of the neurological response to ethanol [2,3], the homeostatic development of dependence [4-6], withdrawal symptoms [7-9], and behavior [10]. The dynamic regulation and disturbances that affect excitatory neurotransmission are of fundamental importance to understanding the mechanisms behind the development of and withdrawal from ethanol dependence. Of particular interest is the ionotropic *N*-methyl-D-aspartate (NMDA) receptor (NMDAR). NMDARs are selectively targeted by ethanol and are an important neurochemical component of dependence and withdrawal [5,11-13]. It has been hypothesized that long-term alcohol abuse leads to mal-adaptive alterations and dysregulation of NMDARs in an attempt to maintain glutamatergic homeostasis [1,5,10,12-15]. These adaptations are such that normal function is only possible in the presence of ethanol [14], and the subsequent cascade of neurotransmitters may be one mechanism by which the physical [1,5,14,15] and behavioral symptoms [10,12,13] of ethanol dependence and withdrawal are manifested.

Interestingly, the number, composition, and location of NMDARs in neurons are not static [16,17], but rather respond to changes in neuronal activity and sensory stimuli in a dynamic manner. It has been suggested that short-term inhibition leads to rapid changes in activity that are dependent on the phosphorylation of NR2 subunits by striatal-enriched tyrosine phosphatase [18-20]. Long-term exposure to ethanol instead results in chronic glutamatergic inhibition and has been shown to lead to compensatory increases in the number, density, and composition of NMDARs at a cellular and synaptic level [3,5,6,21-23]. These compensatory changes in the subunit composition and the quantity of receptors at the surface of the neuron have been shown to be controlled by three activity-dependent mechanisms: insertion, internalization, and lateral movement [17]. Additional receptors may be synthesized and inserted at the synapse [17]. Alternatively, additional receptors may be recruited from some pool of available receptors, either from within the cell or from the local extracellular region [7,24]. These conceptualizations are supported by an observed increase in NR2B subunit mRNA as a response to ethanol treatment [2,21] and an increase in NR1 subunit polypeptide levels in response to chronic ethanol exposure despite unchanged levels of mRNA [25].

When ethanol is removed from the system and the ethanol-induced inhibitory effects decay, overstimulation of NMDARs and subsequent excitotoxic hyperactivity are observed [1,26]. This excessive stimulation can result in a cascade of events that lead to excitotoxic cell death, delayed neuronal degeneration, withdrawal seizures, and increased activity and sensitivity of NMDARs [7,23,27]. Introduction of NMDA during this withdrawal period exacerbates the stimulation of NMDARs and also the seizure symptoms, while antagonists like Dizocilpine (MK-801) and ethanol decrease the frequency of seizures [28,29].

We posit that the neuroadaptive changes in composition, function, and quantity of NMDARs are controlled by a negative feedback loop in which glutamatergic activity is maintained by changes in the number of NMDARs at the synapse. When regularly exposed to pharmacologically active concentrations of ethanol, this control leads to increased tolerance for and dependence on ethanol in order to achieve normal brain function [8]. In our previous work, we developed a mathematical model of a synthesis-only negative feedback control hypothesis [30,31] in which the NMDAR population could only be controlled by synthesizing and inserting new receptors at the synapse. However, recent experimental evidence suggests that receptor synthesis cannot explain the dynamic regulation of NMDA receptors [24,32]. Therefore, this study focuses on the development and implementation of a mathematical representation of a relocation-based control scheme in which neuronal activity is controlled by movement of receptors to and from the synapse and extrasynaptic regions. Ultimately, we evaluate the severity of predicted withdrawal following various alcohol consumption patterns in order to generate testable hypotheses.

## Results and Discussion

### A control system model of NMDAR regulation

In the hopes of better understanding biological responses to alcohol dependence and withdrawal at a systemic level, we developed a mathematical model to describe the dynamics of NMDARs in response to ethanol where we considered the excitatory neurotransmission process as a negative feedback control system. As described in the Methods section, we propose a composite controller with two active components: an activity controller that maintains synaptic activity by sending additional receptors to the synapse, and a density controller that moderates the population of NMDARs at the synapse by removing active, unblocked receptors from the synapse. Together, these controllers function to maintain a constant number of active synaptic receptors in the face of disturbances, such as inhibition of receptors by ethanol.

**Figure 1:**
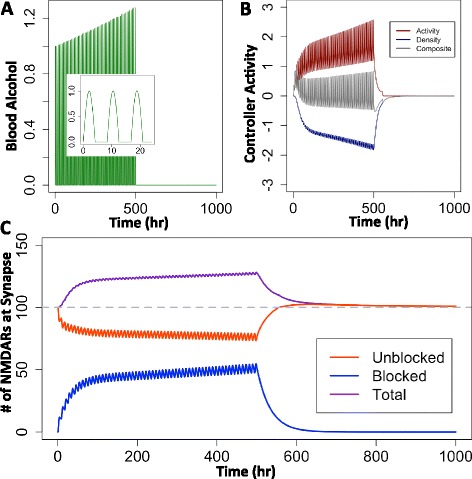
Simulated model response to alcohol consumption. **(A)** The model with Parameter Set A was simulated with a gradually growing dimensionless alcohol input that approximates three alcohol consumption peaks per day (as shown in the 24-hour inset) and an abrupt withdrawal after 500 hours. **(B)** The resultant controller activity, expressed as number of NMDARs translocated to the synapse per hour. In response to the changing levels of active, unblocked NMDARs at the synapse, the activity controller (dark red) moves NMDARs from the extrasynaptic pool to the synapse, while the density controller (navy blue) removes NMDARs from the synapse. The overall, composite controller activity is shown in grey. **(C)** Dynamics of unblocked (red), blocked (blue), and total (purple) NMDARs at the synapse in response to the alcohol profile shown in Panel A.

When simulated with the alcohol profile given in Equation 15 (Figure 1A), the model described in the Methods section with Parameter Set A (Table 1) produced results (Figure 1) that are qualitatively consistent with experimental data in four distinct ways: (1) the synaptic NMDAR population increases with alcohol (Figure 1C) [2,3,5,7,17,21,28,29]; (2) alcohol consumption paradigms affect the severity of outcomes [3,33]; (3) an excitotoxic withdrawal response is observed [1,4,7,13]; and (4) NMDAR populations return to normal levels over time [28].

**Table 1:** **Controller parameter sets**

**Parameter**	**Set A**	**Set B**	**Set C**	**Set D**	**Set E**
*y*_*max1*_ *(rec/hr)*	25	25	25	25	25
*n*_*1*_	2	2	**4**	2	**4**
*a*_*x*_ *(rec)*	50	50	50	**25**	**25**
*a*_*z*_ *(rec)*	50	50	50	**25**	**25**
*k*_*a*_	0.5	0.5	0.5	0.5	0.5
*y*_*max2*_ *(rec/hr)*	25	**12.5**	25	25	25
*n*_*2*_	2	2	**4**	2	**4**
*a*_*2*_ *(rec)*	100	100	100	**50**	**50**
**Figure(s)**	1,2^*^	3A	3B	3C	3D, 4, 5

**Figure 2:**
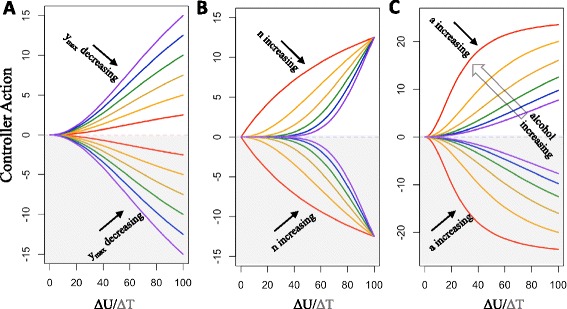
Steady state controller actions for various primary parameter alterations. The number of NMDARs translocated to the synapse per hour by each controller with various values of *y*_*max*_ **(A)**, *n* **(B)**, and *a* **(C)**. In each panel, the action of the activity controller is positive, changing in response to Δ*U*, whereas the action of the density controller is negative and changes according to Δ*T*. Parameter values were altered in common intervals across both controllers. For **(A)**, *y*_*max*_ = 5 (red), 10 (orange), 15 (yellow), 20 (green), 25 (blue), and 30 (purple) receptors/hour. For **(B)**, *n* = 1 (red), 2 (orange), 3 (yellow), 4 (green), 5 (blue), and 6 (purple). For **(C)**, *a* = 25 (red), 50 (orange), 75 (yellow), 100 (green), 125 (blue), and 150 (purple) receptors. Parameter values not explicitly changed are those of Parameter Set A. Increased alcohol concentration decreases the value of *a*_*1*_, as shown by the open arrow in **(C)**.

Values of parameters used for various analyses and the figures associated with the corresponding analyses. Bolded numbers highlight differences from Parameter Set A. ^*^: For each panel of Figure 2, the indicated parameter was altered from its nominal value in Parameter Set A.

Here, the explicit set point was fixed at an arbitrary value of 100 NMDARs at the synapse, as indicated by the dashed line in Figure 1C. As alcohol molecules block the active, unblocked receptors, the composite controller attempts to maintain the set point value by receptor translocation to (activity controller, dark red) and from (density controller, navy blue) the extrasynaptic pool of receptors (Figure 1B).

### A tunable composite controller

The proposed composite controller consists of six primary parameters: *y*_*max1*_, *n*_*1*_, *a*_*1*_*(A)*, *y*_*max2*_, *n*_*2*_, and *a*_*2*_, where *a*_*1*_ is a function of alcohol concentration involving three secondary parameters: *a*_*x*_, *a*_*z*_, and *k*_*a*_ (Equations 10–12). The steady state controller activity varies with changes in the primary parameters as shown in Figure 2. Increases in alcohol concentration enhance the actions of the activity controller through its effect on *a*_*1*_ (Equation 12). This provides a mechanism for incorporating alterations in the apparent set point during long-term exposure to alcohol, which is one hypothesis for the development of alcohol dependence [1,7,8,21,25].

**Figure 3:**
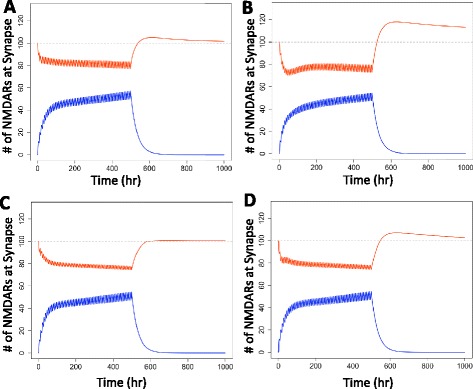
The magnitude and duration of predicted excitotoxicity varies with controller configuration. In response to the alcohol input of Equation 15 (shown in Figure 1A), the number of blocked (blue) and unblocked (red) NMDARs at the synapse varies with controller configuration. Controller parameters are listed in Table 1: **(A)** Parameter Set B, **(B)** Parameter Set C, **(C)** Parameter Set D, **(D)** Parameter Set E. Excitotoxicity is inferred when the number of unblocked receptors at the synapse is greater than 100 (the arbitrarily defined explicit set point).

Clinical reports suggest a wide variety among individuals’ neuroexcitatory activity during alcohol dependence and withdrawal based on genetics [34], gender [3], and behavior [33]. Our composite controller is tunable to approximate a range of activity. Figure 3 shows the simulated results for four alternative controller configurations (Parameters Sets B-E, Table 1) responding to the same alcohol input (Equation 15). The magnitude and duration of the predicted excitotoxicity following withdrawal varies considerably among these alternative configurations.

### Consumption patterns leading to dependence Do Not influence predicted withdrawal severity

**Figure 4:**
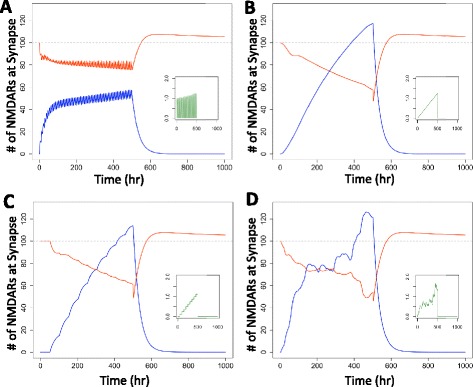
The specific pattern of alcohol consumption prior to withdrawal does not affect withdrawal severity. The number of unblocked (red) and blocked (blue) NMDARs at the synapse for various dimensionless alcohol consumption patterns (green insets). For periodic increases **(A)**, constantly increasing levels **(B)**, incremental increases **(C)**, and randomly distributed dimensionless alcohol levels **(D)**, the excitotoxicity after withdrawal is relatively uniform.

Clinicians have no control over the alcohol consumption pattern that leads to alcohol dependence, and frequently the pattern is unknown. In order to gauge the relative import of specific consumption patterns on predicted withdrawal severity, we simulated four alcohol consumption patterns. We selected Parameter Set E for these investigations, because, as shown in Figure 3D, this configuration led to moderately severe predicted withdrawal upon cessation of alcohol input. As shown in Table 2 and the insets of Figure 4, all four proposed alcohol inputs involve a gradually increasing dimensionless alcohol level that ends abruptly after 500 hours. The profiles vary in consumption pattern from an idealized linear increase to a randomized pattern of intake. The simulated results of NMDAR levels at the synapse are shown in Figure 4. Interestingly, the severity of alcohol withdrawal, as quantified by the area under the curve and the maximum number of unblocked receptors at the synapse, does not change appreciably (<10%, Table 2). In fact, as long as the consistency of exposure, peak ethanol concentration, and withdrawal profile are similar, the severity of withdrawal is largely the same. This suggests that although withdrawal severity differs considerably with controller parameters (Figure 3, akin to different activities in different individuals), the specific pattern of alcohol consumption with a given duration prior to withdrawal does not affect predicted withdrawal severity (Figure 4, Table 2).

**Table 2:** **Measures of withdrawal severity for the four alcohol dependence profiles tested**

**Alcohol profile**	**Withdrawal profile**	**Area under curve**	**Unblocked max**
Equation #15	$$ \left\{\begin{array}{c}\hfill t\ge 500\hfill \\ {}\hfill A(t)=0\hfill \end{array}\right\} $$	2893	107.77
*A*(*t*) = 0.0025*t*	$$ \left\{\begin{array}{c}\hfill t\ge 500\hfill \\ {}\hfill A(t)=0\hfill \end{array}\right\} $$	2743	107.77
$$ \left\{\begin{array}{c}\hfill A\left(t=0\right)=0\hfill \\ {}\hfill Incremental\ increase\ of\hfill \\ {}\hfill 0.125\ every\ 50\ hours\hfill \end{array}\right\} $$	$$ \left\{\begin{array}{c}\hfill t\ge 500\hfill \\ {}\hfill A(t)=0\hfill \end{array}\right\} $$	2743	107.77
$$ \left\{\begin{array}{c}\hfill p\le 0.1\ A(t)=A\left(t-1\right)+0.1\hfill \\ {}\hfill p>0.1\ A(t)=A\left({t}_{last}\right){e}^{-0.01\Big({t}_{last}-t}\hfill \end{array}\ \right\} $$ Where p is a randomly generated probability and *t*_*last*_ is the time alcohol was last consumed.	$$ \left\{\begin{array}{c}\hfill t\ge 500\hfill \\ {}\hfill A(t)=0\hfill \end{array}\right\} $$	2680	107.77

The corresponding dynamic responses are shown in Figure 4.

### Alcohol consumption during withdrawal affects predicted withdrawal severity

During withdrawal, administration of ethanol and other NMDAR antagonists has been shown to decrease the severity of withdrawal symptoms in humans and rodents and decrease excitotoxicity in cultured neurons [28,29]. Unfortunately, the frequency and dosage of NMDAR antagonist administration in in-patient settings is driven symptomatically and administered reactively. Our model provides the opportunity to try any withdrawal pattern risk-free and evaluate the predicted withdrawal severity, even patterns that are not easily implemented clinically. This could lead to proactive administration of antagonist thereby preventing symptoms and excitotoxic damage.

**Figure 5:**
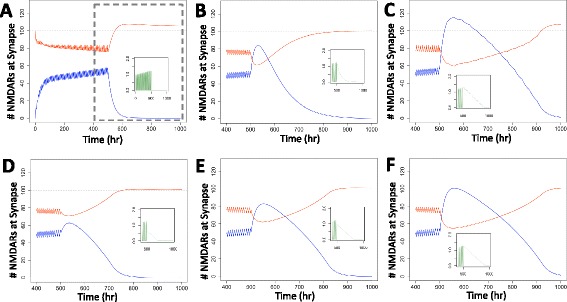
Alcohol levels during withdrawal affect the severity of withdrawal. The number of unblocked (red) and blocked (blue) NMDARs at the synapse in response to various withdrawal patterns (green insets). The full time course is shown in **(A)**. The remaining panels show only *t* > 400 hr, the region inside the dashed box in **(A)**. The response is identical in all withdrawal schemes at *t* < 500 hr. Withdrawal was initiated at *t* = 500 hr in various patterns: **(A)** abrupt and complete cessation; **(B)** exponential decrease of alcohol; **(C)** gradual incremental decreases; **(D)** constant decrease from ½ maximum alcohol level; **(E)** constant decrease from ¾ maximum alcohol level; **(F)** constant decrease from maximum alcohol level.

Whereas the alcohol pattern leading to dependence did not influence the quantified measures of withdrawal appreciably, the alcohol pattern during withdrawal has a large impact on these measures. For the pre-withdrawal alcohol input given in Equation 15 for *t* < 500 hr, Figure 5 shows the predicted synaptic NMDAR populations during six withdrawal regimes. These regimes include complete cessation (Figure 5A), exponential decay (Figure 5B), step-wise decreases (Figure 5C), and linearly decreasing alcohol profiles with various initial amounts (Figures 5D-F). The quantified severity of withdrawal is shown in Table 3. As expected, additional alcohol present during the withdrawal period decreases the severity of withdrawal, primarily in terms of the area under the curve. The maximum number of unblocked receptors observed is fairly consistent during all withdrawal regimes tested; we expect this to be a function of the controller parameters, which were constant for all withdrawal regimes tested here.

**Table 3:** **Measures of withdrawal severity for the six withdrawal profiles tested**

**Alcohol profile**	**Withdrawal Profile**	**Area under curve**	**Unblocked max**
Equation 15	$$ \left\{\begin{array}{c}\hfill t\ge 500\hfill \\ {}\hfill A(t)=0\hfill \end{array}\right\} $$	2883	107.77
Equation 15	$$ \left\{\begin{array}{c}\hfill t\ge 500\hfill \\ {}\hfill A(t)={A}_{max}{e}^{-0.015\left(t-{t}_{withdraw}\right)}\hfill \end{array}\right\} $$	54.1	100.64
Equation 15	$$ \left\{\begin{array}{c}\hfill t\ge 500\ \hfill \\ {}\hfill A(t)={A}_{max}\hfill \\ {}\hfill Step\ Down\ 0.1\ every\ 30\ hours\hfill \end{array}\right\} $$	2883	107.66
Equation 15	$$ \left\{\begin{array}{c}\hfill t\ge 500\hfill \\ {}\hfill A(t)=0.5{A}_{max}-0.003\left(t-{t}_{withdraw}\right)\hfill \end{array}\right\} $$	260.5	101.38
Equation 15	$$ \left\{\begin{array}{c}\hfill t\ge 500\hfill \\ {}\hfill A(t)=0.75{A}_{max}-0.003\left(t-{t}_{withdraw}\right)\hfill \end{array}\right\} $$	157.3	101.38
Equation 15	$$ \left\{\begin{array}{c}\hfill t\ge 500\hfill \\ {}\hfill A(t)={A}_{max}-0.003\left(t-{t}_{withdraw}\right)\hfill \end{array}\right\} $$	19.3	101.11

The corresponding dynamic responses are shown in Figure 5.

We note that the sudden drop in unblocked receptors and peak in blocked receptors observed in the ramp, step down, and exponential decay profiles may seem counterintuitive; a peak in unblocked receptors is expected to coincide with observed withdrawal symptoms. However, this behavior is due to the shift from periodic to sustained alcohol levels. When the alcohol level deviates between large values and zero (as it does at *t* < 500 hr), the controller activity mimics these changes. Consistent controller response, however, leads to a large increase in the number of synaptic receptors, but the high ethanol level initiating this consistent response means that the receptors are quickly blocked, and become unblocked gradually as alcohol level diminishes. This suggests that even if it were possible to maintain a non-zero alcohol level during in-patient withdrawal, the effects on NMDAR-mediated neuroexcitatory processes would not be favorable.

### Limitations and caveats

We are unaware of any experimental data measuring the translocation of NMDARs in human brain tissue. Therefore, the kinetics shown here are only hypothetical realizations of our control system hypothesis. Wherever possible, we have attempted to use dimensionless (e.g. alcohol level) or easily scalable (e.g. *y*_*max*_) functions and parameters so that the model could be adapted easily to fit experimental data.

Furthermore, the predicted control actions do not reveal mechanistic information. For example, it has been established that NMDAR subunit composition changes in response to alcohol [6,17,29,35], promoting a removal of NMDARs from the synapse. In our model, this is represented in the bulk sensing of blocked NMDARs and removal of receptors by the density subcontroller.

Finally, we recognize that the severity of alcohol withdrawal cannot be predicted by the levels of unblocked NMDARs alone. For example, the neuroinhibitory system (especially GABA_A_ receptors) has been implicated in the brain’s response to alcohol [1,36,37]. A complete representation of withdrawal would require incorporation of these additional systems. However, given the excitotoxic nature of the most detrimental symptoms of alcohol withdrawal (delirium tremens, seizures, etc.), we have focused our efforts on describing the neuroexcitatory effects of alcohol via NMDARs.

## Conclusions

In this work, we have developed a computational model based on a negative feedback control system hypothesis of NMDAR regulation at the synapse in the presence of alcohol. We posit that NMDARs are translocated from an extrasynaptic pool to the synapse by an activity subcontroller in order to maintain a set number of unblocked, active NMDARs at the synapse. Simultaneously, NMDARs are removed from the synapse by a density subcontroller to maintain a constant density of total NMDARs at the synapse. The composite action of the two subcontrollers aims to maintain glutamatergic signaling even when NMDARs are blocked by ethanol molecules. The proposed composite controller is tunable for a variety of individual responses or to match any future experimental data, and the resulting model produces results consistent with qualitative experimental data describing the biophysical causes of both dependence and withdrawal across a range of values for controller parameters.

Our results suggest that withdrawal severity is not influenced by the manner in which alcohol dependence is achieved, provided that the state of dependence is similar. This suggests that for a particular individual (analogously, a particular set of controller parameters), the prediction of withdrawal severity depends on the characterization of the current state of dependence (frequency, quantity, and duration of alcohol consumption) and the specific parameters of the individual’s NMDAR controller activity.

The severity of alcohol withdrawal is, however, influenced by the alcohol input during withdrawal. This is consistent with experimental results that showed that administration of NMDAR antagonists such as ethanol reduce the negative effects of alcohol withdrawal [8], while the administration of NMDAR agonists such as NMDA increase the severity of withdrawal symptoms [7,27]. The results of this work show that the most effective means of reducing excitotoxicity involve exacerbating the response with increased total alcohol and then carefully decreasing alcohol levels over a prolonged period of time. This is not likely to be a viable option clinically. The model provides tremendous flexibility for conducting *in silico* investigations of alternative withdrawal profiles in order to gain a better understanding of how changes in the dependence and withdrawal profiles can affect the outcomes of excitotoxic withdrawal and long-term changes to system dynamics and to generate testable hypotheses.

## Methods

### The feedback control system and governing equations

**Figure 6:**
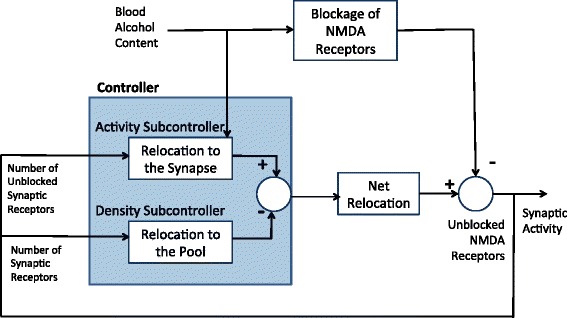
Proposed control system block diagram representation. Synaptic activity resulting from the glutamatergic signaling of NMDARs is controlled via a dual-action composite controller (blue box). The activity subcontroller of this composite controller translocates NMDARs from an extrasynaptic pool to the synapse in response to deviations from the explicit set point number of unblocked synaptic NMDARs. The activity of this controller is also modulated by the alcohol level. The density subcontroller removes unblocked NMDARs from the synapse according to the deviation in total NMDARs (blocked and unblocked) from the set point. The combined activity of the two subcontrollers contributes to a net relocation that alters the number of unblocked synaptic NMDARs. Unblocked NMDARs can also be blocked by alcohol. Measurements of the number of unblocked NMDARs and the total number of synaptic NMDARs are then returned to the controller via a negative feedback loop.

In this study, we consider a compensatory negative feedback control mechanism as a response to ethanol-induced NMDAR inhibition, as shown in the control system block diagram in Figure 6. The ultimate control objective here is to maintain normalized brain function in the presence of ethanol by maintaining a constant level of unblocked NMDARs (*U*) at the synapse.

When ethanol is introduced to the system, unblocked receptors (*U*) become blocked (*B*) by alcohol (*A*) according to a reversible reaction with elementary kinetics, as shown in Equation 1:

1 $$ U+A\underset{k_2}{\overset{k_1}{\rightleftarrows }}B $$

where *k*_*1*_ = 0.05 hr^−1^ and *k*_*2*_ = 0.03 hr^−1^. The number of NMDARs is somehow sensed or measured by the cell in a process that we have assumed to have a perfect gain and negligible dynamics, akin to most biological sensors. This information is then processed by a two-part composite controller, whose combined action, *C*_*T*_*(t)*, changes the number of unblocked receptors (*U*) at the synapse. The overall changes in *U* and *B* are therefore governed by Equations 2 and 3:

2 $$ \frac{dU(t)}{dt}=-{k}_1A(t)U(t) + {k}_2B(t)+{C}_T(t) $$

3 $$ \frac{dB(t)}{dt}={k}_1A(t)U(t) - {k}_2B(t) $$

### Development of a composite controller

In order to achieve bi-directional action, the composite controller must be able to move receptors to and from the synapse. Previous work by Staehle et al. considered a unidirectional controller with sigmoidal shaped steady state characteristic activity to move unblocked NMDARs to the synapse [31]. Without a mechanism for reducing the number of receptors at the synapse, this controller was unable to capture expected behavior during withdrawal. Therefore, in this study, we have developed a dual-mode, bi-directional composite controller for modulation of synaptic unblocked NMDARs. One subcontroller inserts new unblocked NMDARs from an extrasynaptic “pool” based on current levels of synaptic unblocked NMDARs in an effort to maintain a defined population of unblocked receptors; this will be referred to henceforth as the activity controller. The second subcontroller removes unblocked NMDARs from the synapse in an effort to maintain a fixed number of NMDARs at the synapse. This controller does not discriminate whether the synaptic receptor is blocked or unblocked in its assessment of synaptic density, but only removes active, unblocked NMDARs from the synapse. NMDAR receptor trafficking is activity dependent [17] and consequently the trafficking and localization of receptors blocked by ethanol is inhibited [3,38]. We have therefore assumed that inhibited receptors are inaccessible for the molecular mechanisms responsible for this. The second subcontroller will be referred to as the density controller. The dual-mode construction yields two subcontrollers of the following forms, where *C*_*1*_ controls relocation to the synapse by the activity controller, *C*_*2*_ controls relocation from the synapse by the density controller, and *C*_*T*_ represents the net control action:

4 $$ T(t)=U(t)+B(t) $$

5 $$ {C}_1(t) = {y}_{max1}\left(\frac{U{(t)}^{n_1}}{U{(t)}^{n_1}+{a}_1{(A)}^{n_1}}\right) $$

6 $$ {C}_2(t)=-{y}_{max2}\left(\frac{T{(t)}^{n_2}}{T{(t)}^{n_2}+{a_2}^{n_2}}\right) $$

7 $$ {C}_T(t)={C}_1(t)+{C}_2(t) $$

The control laws hypothesized for this system (Equations 5 and 6) are based on similar mathematical studies of steady state controller action in biological systems [39-41], and it is hypothesized that this sigmoidal formulation captures the physical limitations of biological processes. In these descriptions, *y*_*max*_ represents the maximum controller action, while *a* and *n* are position and shape parameters that shift steady state controller activity plots and change the curvature, respectively.

The controller activity formulation of Equations 5–7 is complicated by the fact that the two subcontrollers cause significant deviation in the implicit set point. Changes to the parameters of either controller shifts the number of receptors at which the controller actions are balanced, which is the effective set point for the system. Manipulating parameters to achieve the desired set point is feasible when only one controller is involved, but with the additional complexity of a second subcontroller, an explicit set point is required. The formulation utilizing an explicit set point is provided in Equations 8–11.

8 $$ \varDelta U(t)={U}_{Desired}-U(t) $$

9 $$ \varDelta T(t)={U}_{Desired}-T(t)={U}_{Desired}-U(t)-B(t) $$

10 $$ {C}_1(t)={y}_{max1}\left(\frac{\varDelta U{(t)}^{n_1}}{a_1{(A)}^{n_1}+\varDelta U{(t)}^{n_1}}\right) $$

11 $$ {C}_2(t)=-{y}_{max2}\left(\frac{\varDelta T{(t)}^{n_2}}{{a_2}^{n_2}+\varDelta T{(t)}^{n_2}}\right) $$

In this formulation, the controller activity is based upon the deviation of the measured value from the explicit set point. For this study, we have defined the explicit set point, *U*_*Desired*_, as 100 receptors. This value is arbitrary and can be scaled according to biochemical data. We have also assumed that the population of NMDARs in the “pool” is never limiting and thus the calculated *C*_*1*_ controller activity is always realizable. This is assumption is valid as long as both controllers are active, and would need to be revisited for scenarios in which the activity of one controller dominates (e.g. approximations of co-morbid disease states). Furthermore, both controllers are constrained in line with biophysical limitations on their control actions: *C*_*1*_ has no activity if *U(t)* > *U*_*Desired*_ and *C*_*2*_ has no activity if *T(t)* < *U*_*Desired*_*.*

Finally, we posit that the desired number of unblocked NMDARs at the synapse shifts in response to alcohol intake. Thus, we redefined the position parameter of the activity subcontroller, *a*_*1*_, to be a function of blood alcohol content. In general, with smaller values of *a*_*1*_, small changes in *U* create large changes in controller output. As *a*_*1*_ increases, larger deviations in *U* are required to obtain the same controller action. Defining this parameter as a function of alcohol also allows the relative actions of the activity and density subcontrollers to drift with alcohol input. To capture the alcohol dependency, we have defined *a*_*1*_ as an Arrhenius function deviation from an initial value, as shown in Equation 12.

12 $$ {a}_1(A)={a}_x+{a}_z{e}^{-{k}_aA(t)} $$

The position parameter is defined in this manner so that the controller response is quick when ethanol content is low but requires larger deviations when alcohol level increases.

In the development of this control scheme, we have made a number of assumptions about the glutamatergic neurotransmission system. First, we assume that the overall glutamate load of these neurons is reasonably constant such that ethanol is the only stimulus modulating the number of NMDARs required at the synapse. This allows the disturbances to the system to be described as a single function representing alcohol intake. Second, we assume that synaptic activity is primarily a function of receptor population and density; the receptor population herein can therefore be considered as a homogenous population with characteristics of the average composition and activity of synaptic NMDARs.

### Simulation

Two methods were utilized to simulate the model. First, a numerical integration of Equations 2 and 3 was conducted using Euler’s method in Visual Basic and Microsoft Excel® with a Δ*t* of 0.1 hrs. This method is required for alcohol intake patterns with discontinuities. The system was also simulated by differentiating Equations 10 and 11 to obtain Equations 13 and 14, and then solving the system of differential equations with an ordinary differential equation solver (ode15s) in MATLAB®.

13 $$ \frac{d{C}_1}{dt}=\frac{-{y}_{max1}{n}_1}{{\left({a}_1{(A)}^{n_1}+\varDelta U{(t)}^{n_1}\right)}^2}\left({a}_1{(A)}^{n_1}\varDelta U{(t)}^{n_1-1}\frac{dU}{dt}+{k}_a{a}_z{a}_1{(A)}^{n_1-1}\varDelta U{(t)}^{n_1}{e}^{-{k}_aA(t)}\frac{dA(t)}{dt}\right) $$

14 $$ \frac{d{C}_2}{dt}=\frac{y_{max2}{n}_2}{{\left(\varDelta T{(t)}^{n_2}+{a_2}^{n_2}\right)}^2}\left[{a_2}^{n_2}\varDelta T{(t)}^{n_2-1}\left(\frac{dU}{dt}+\frac{dB}{dt}\right)\right] $$

### Alcohol input functions

Alcohol was initially represented by a chronically increasing sinusoid with fixed periodicity and abrupt withdrawal at a specified time *t*_*w*_, as described in Equation 15:

15 $$ A(t)=\left\{\begin{array}{c}\hfill 0\hfill \\ {}\hfill 0\hfill \\ {}\hfill Zsin(pt) \exp (gt)\hfill \end{array}\right.\ for\kern0.5em \begin{array}{c}\hfill \sin (pt)<0\hfill \\ {}\hfill t>{t}_w\hfill \\ {}\hfill otherwise\hfill \end{array} $$

The periodicity parameter, *p*, describes the frequency of alcohol consumption, *g* describes the growth of ethanol consumption over time, *t*_*w*_ is the time at which the desired withdrawal profile is imposed, and *Z* provides a scaling factor for normalizing the dimensionless alcohol level. As in our previous work [30,31], we used *p* = 0.75 hr^−1^, *g* = 5x10^−4^ hr^−1^, *t*_*w*_ = 500 hr, and *Z* = 1. Additional alcohol input functions were developed to investigate the system response to various alcohol dependence and withdrawal paradigms. These functions are stated and/or illustrated in the Results and Discussion.

### Assessment of predicted withdrawal severity

A primary goal of this study was to identify whether changes in dependence or withdrawal alcohol profile affect the severity of withdrawal symptoms. In order to quantify and compare the predicted withdrawal severity under various circumstances with our theoretical model, we calculated two measures: area under the curve and unblocked max. The area under the curve was calculated as an approximate integral between the explicit set point and the actual unblocked receptor curve following the initiation of withdrawal. This calculation was conducted using a midpoint approximation for all points after the initiation of withdrawal where the number of unblocked NMDARs was above the set point. The time step for this approximation was 0.1 hours. This metric provides insight into the severity of lasting changes in functionality and the number of synaptic unblocked NMDARs. The peak value of unblocked synaptic NMDARs was determined as the maximum number of NMDARs after the initiation of withdrawal. The peak value provides insight into the peak glutamatergic activity, or the extent of excitotoxicity during withdrawal.

## Abbreviations

NMDA: *N*-methyl-D-aspartate
NMDAR: *N*-methyl-D-aspartate Receptor
